# Inhibition of Hsp90 Counteracts the Established Experimental Dermal Fibrosis Induced by Bleomycin

**DOI:** 10.3390/biomedicines9060650

**Published:** 2021-06-07

**Authors:** Hana Štorkánová, Lenka Štorkánová, Adéla Navrátilová, Viktor Bečvář, Hana Hulejová, Sabína Oreská, Barbora Heřmánková, Maja Špiritović, Radim Bečvář, Karel Pavelka, Jiří Vencovský, Jörg H. W. Distler, Ladislav Šenolt, Michal Tomčík

**Affiliations:** 1 Institute of Rheumatology, 12800 Prague, Czech Republic; storkanova@revma.cz (H.Š.); storkanoval@revma.cz (L.Š.); navratilova@revma.cz (A.N.); becvarv@revma.cz (V.B.); hulejova@revma.cz (H.H.); oreska@revma.cz (S.O.); spiritovic@revma.cz (M.Š.); becvar@revma.cz (R.B.); pavelka@revma.cz (K.P.); vencovsky@revma.cz (J.V.); senolt@revma.cz (L.Š.); 2 Department of Rheumatology, First Faculty of Medicine, Charles University, 12800 Prague, Czech Republic; 3 Department of Physiotherapy, Faculty of Physical Education and Sport, Charles University, 16252 Prague, Czech Republic; hermankova@revma.cz; 4 Department of Internal Medicine III and Institute for Clinical Immunology, University of Erlangen-Nuremberg, 91054 Erlangen, Germany; joerg.distler@uk-erlangen.de

**Keywords:** heat shock protein 90, systemic sclerosis, established dermal fibrosis, treatment

## Abstract

Our previous study demonstrated that heat shock protein 90 (Hsp90) is overexpressed in the involved skin of patients with systemic sclerosis (SSc) and in experimental dermal fibrosis. Pharmacological inhibition of Hsp90 prevented the stimulatory effects of transforming growth factor-beta on collagen synthesis and the development of dermal fibrosis in three preclinical models of SSc. In the next step of the preclinical analysis, herein, we aimed to evaluate the efficacy of an Hsp90 inhibitor, 17-dimethylaminoethylamino-17-demethoxygeldanamycin (17-DMAG), in the treatment of established experimental dermal fibrosis induced by bleomycin. Treatment with 17-DMAG demonstrated potent antifibrotic and anti-inflammatory properties: it decreased dermal thickening, collagen content, myofibroblast count, expression of transforming growth factor beta receptors, and pSmad3-positive cell counts, as well as leukocyte infiltration and systemic levels of crucial cytokines/chemokines involved in the pathogenesis of SSc, compared to vehicle-treated mice. 17-DMAG effectively prevented further progression and may induce regression of established bleomycin-induced dermal fibrosis to an extent comparable to nintedanib. These findings provide further evidence of the vital role of Hsp90 in the pathophysiology of SSc and characterize it as a potential target for the treatment of fibrosis with translational implications due to the availability of several Hsp90 inhibitors in clinical trials for other indications.

## 1. Introduction

Systemic sclerosis (SSc, scleroderma) is a rare chronic autoimmune connective tissue disease of a complex etiopathogenesis characterized by vasculopathy, dysregulation of the immune system, and tissue fibrosis [1]. Fibrosis of the skin and internal organs, such as the lungs, gastrointestinal tract, heart, and kidneys, is the most characteristic feature of SSc [1]. During the course of the disease, the abnormally activated innate and acquired immune system affects resident fibroblasts, which are the critical cellular contributors to tissue fibrosis in SSc [1,2]. Chronically activated fibroblasts release an excessive amount of collagen and extracellular matrix (ECM) proteins, which leads to severe dysfunction of the affected tissues [2,3]. Multiple lines of evidence suggest that transforming growth factor-beta (TGF-β) plays a crucial role in the activation of fibroblasts and the development of tissue fibrosis in SSc [3]. TGF-β is able to induce transdifferentiation of fibroblasts into contractile cells called myofibroblasts, the microfilaments of which consist of alpha-smooth muscle actin (aSMA) and non-muscle myosin type II [3,4]. Myofibroblasts increase the production of collagen, fibronectin, proteoglycans, and other components of the ECM [3,5]. Moreover, TGF-β induces heterotetramerization of TGF-β-receptor type I (TβRI) and II (TβRII) and leads to activation of several intracellular pathways, particularly the ones mediated by small mothers against decapentaplegic homolog 3 (Smad3) and other kinases [3,5]. However, myofibroblasts have also been attributed an immunomodulatory role since they express interleukin (IL)-1, IL-6, IL-8, and monocyte chemoattractant protein 1 (MCP-1, CCL2) [6,7]. Despite recent substantial advancements shedding light on the pathophysiology of tissue fibrosis in SSc [8], an effective and available clinical treatment with disease-modifying and survival-improving effects has yet to be determined [9]. 

Heat shock protein 90 (Hsp90) belongs to the ubiquitous molecular chaperone family of heat shock proteins (Hsp), which are produced by a wide range of cells under conditions of cellular stress [10]. Hsp play an important role in protein–protein interactions, such as recognizing and binding to non-natively folded proteins, assisting in achieving their proper conformation, and preventing against their irreversible degradation and activation [11,12]. Hsp90 interacts with a wide range of substrate proteins, including kinases, transcription factors, steroid hormone receptors, and E3 ubiquitin ligases [13]. These client proteins are essential for many biological processes such as cell cycle and growth, apoptosis, cytoskeletal rearrangement, and many others [13,14,15]. Furthermore, Hsp90 mediates the activation and maturation of antigen-presenting cells and the induction of proinflammatory cytokines [16,17]. Hsp90 has been demonstrated to participate in autoimmune response, oncogenesis, viral infections, and neurodegenerative diseases [13,16,18,19,20,21,22]. In addition, Hsp90 contributes to stabilization and activation of TGF-β receptors (TβRI and TβRII) and Src kinases, which are intracellular mediators for profibrotic TGF-β signaling in SSc [23,24,25]. In our recent study, we described increased expression of Hsp90 in the involved skin of patients with SSc, in SSc dermal fibroblasts and in experimental dermal fibrosis in a TGF-β-dependent manner [26]. Furthermore, the inhibition of Hsp90 with a semi-synthetic derivative of geldanamycin, 17-dimethylaminoethylamino-17-demethoxy-geldanamycin (17-DMAG), demonstrated antifibrotic effects in vitro and prevented the development of dermal fibrosis in three preclinical models mimicking various stages of SSc [26]. 

In this study, as the next step of the preclinical analysis, we aimed to evaluate the efficacy of 17-DMAG in the established experimental dermal fibrosis, which better reflects the routine clinical practice, where most SSc patients seen by a rheumatologist have already developed tissue fibrosis. Therefore, we used a modified murine model of bleomycin-induced dermal fibrosis, in which the administration of bleomycin was prolonged to six weeks, and the treatment onset with 17-DMAG was delayed to the last three weeks of the ongoing bleomycin challenge [27,28]. To analyze both antifibrotic and anti-inflammatory properties exerted by 17-DMAG, we used the traditional outcome measures assessing dermal thickness, collagen content, activation of fibroblasts and of TGF-β pathway, including expression of pSmad3, TβRI, and TβRII, as well as markers of local and systemic inflammation, including selected cytokines and chemokines with established proinflammatory roles in the pathogenesis of SSc [1,2,7,29]. Given that several inhibitors of Hsp90 have already been tested in numerous clinical trials for other indications, this study could provide a novel therapy for the treatment of fibrosis in SSc with high translational potential [30,31,32,33]. 

## 2. Methods

### 2.1. Treating Established Bleomycin-Induced Dermal Fibrosis

**Figure 1:**
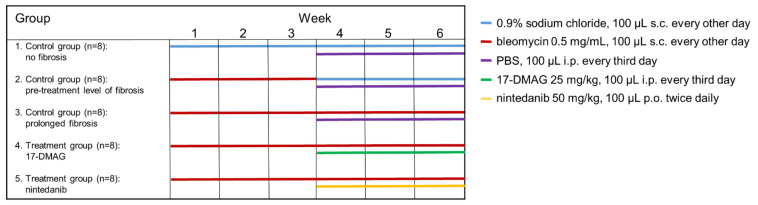
Design of the modified model of bleomycin-induced experimental dermal fibrosis. PBS, phosphate buffered saline; 17-DMAG, 17-dimethylaminoethylamino-17-demethoxy-geldanamycin (inhibitor of heat shock protein 90); s.c., subcutaneously administered; i.p., intraperitoneally administered; p.o., perorally administered.

A modified bleomycin model was used to analyze the efficacy of the Hsp90 inhibitor 17-DMAG in the regression of preestablished fibrosis [28,34,35,36]. Robust dermal fibrosis was first induced by injecting bleomycin for the first three weeks solely without treatment. A three-week course of treatment was then initiated at the start of the fourth week of the experiment, while bleomycin injections were continued for the remaining three weeks of the whole six-week experiment. The outcomes were analyzed six weeks after the first injection of bleomycin [28,34,35,36]. The following five groups of six-week-old male C57BL/6 mice (Velaz, s.r.o., Prague, Czech Republic) were included (Figure 1). The modified bleomycin model was performed as described previously [28,36,37], and can be briefly summarized as follows:

(1) The first control group was administered subcutaneous injections of 100 μL 0.9% NaCl every other day for six weeks, and served as a control for treatment with bleomycin (*n* = 8).
(2) The second control group of mice (*n* = 8) was subcutaneously injected with bleomycin for the first three weeks and with 0.9% NaCl for the last three weeks. The level of achieved dermal fibrosis in this group after the first three weeks of subcutaneous bleomycin injections represents the pretreatment level of established dermal fibrosis.
(3) Dermal fibrosis was induced by subcutaneous injections of bleomycin (Bleomedac, Medac GmbH, Wedel, Germany) dissolved in 0.9% sodium chloride (NaCl, B. Braun Medical s.r.o., Prague, Czech Republic) at a concentration of 0.5 mg/mL [38,39]. One hundred microliters of bleomycin was administered into the defined area of 1 cm^2^ at the upper back every other day for six weeks (*n* = 8 mice) [38]. Vehicle treatment in groups 1–3 was performed with Dulbecco’s Phosphate Buffered Saline (PBS, Lonza, Walkersville, MD, USA), 100 μL intraperitoneally, every third day in the last three weeks of the six-week experiment.
(4) The main treatment group was challenged with bleomycin for six weeks as described above. In the last three weeks of this six-week period, mice (*n* = 8) were treated intraperitoneally every third day with 100 μL of 17-DMAG (InvivoGen, San Diego, CA, USA) at a concentration of 25 mg/kg (5 mg/mL in PBS, Lonza) [26]. The dose of 17-DMAG used in this study had previously been shown to effectively inhibit Hsp90 in vivo [40], and to be well-tolerated in a chronic dose regimen of up to 180 days in vivo [41].
(5) For the control treatment, we chose a small-molecule competitive inhibitor of nonreceptor tyrosine kinases (nRTKs), nintedanib, as an established antifibrotic agent (kindly provided by Boehringer Ingelheim Pharma GmbH & Co.KG, Ingelheim am Rhein, Germany). These mice (*n* = 8) were challenged with bleomycin for six weeks as described above, and in the last three weeks of this six-week period, nintedanib 50 mg/kg (100 μL diluted in deionized water) was administered twice daily perorally [28,42].

This project (reference number AZV 16-33542A) and all animal experiments included were approved by the Ethics Committee of the Institute of Rheumatology in Prague (reference number 5689/2015, approved on 6 June 2015) and the Ministry of Education, Youth and Sports of the Czech Republic (reference number MSMT-9445/2018-7, approved on 5 May 2018). Animal experiments were conducted in accordance with relevant national legislation on the use of animals for research and complied with the commonly accepted 3Rs.

### 2.2. Histological Analysis of Dermal Thickness

Dermal thickness was assessed as described previously [43]. Injected skin areas were excised, then fixed in 4% formalin for 8 h and embedded in paraffin. Five-micrometer-thick sections were stained with hematoxylin–eosin and visualized by a BX53 microscope with a DP80 Digital Microscope Camera and CellSens Standard Software 3.1-Build 21199 (Olympus, Philadelphia, PA, USA) at 100-fold magnification. Dermal thickness was analyzed by two experienced examiners blinded to the treatment by measuring the maximum distance between the epidermal–dermal junction and the dermal–subcutaneous fat junction at four sites in four consecutive skin sections of the lesional skin from each mouse. The mean value for each mouse was used [43]. 

### 2.3. Assessment of the Number of Infiltrating Leukocytes

Infiltrating leukocytes in the lesional murine skin were quantified in hematoxylin and eosin-stained sections as described previously [43]. The assessment of the number of mononuclear/inflammatory cells (at 400-fold magnification) was performed by two experienced examiners blinded to the treatment in the full thickness of the dermis at eight high-power fields from different tissue sites in four consecutive skin sections of the lesional skin from each mouse. The mean value for each mouse was used [43]. 

### 2.4. Hydroxyproline Assay

The collagen content in lesional skin samples was determined by hydroxyproline assay as described previously [44]. Briefly, each punch biopsy specimen was digested in 6 M HCl for three hours at 120 °C, and the pH of the samples was adjusted to 7 with 6 M NaOH. Afterward, samples were mixed with 0.06 M chloramine T and incubated for 20 min at room temperature. Subsequently, 20% p-dimethylaminobenzaldehyde and 3.15 M perchloric acid were added, and samples were incubated for an additional 20 min at 60 °C. The absorbance was measured at 557 nm with a microplate spectrophotometer (SUNRISE; Tecan, Grödig, Austria) [43]. Two punch biopsies from the central third of the lesional skin of each mouse were analyzed, and the hydroxyproline content was normalized to the dry weight of each biopsy. The mean value was used. For direct visualization of collagen fibers, trichrome staining was performed using the Blue Masson’s Trichrome Stain Kit (Sigma-Aldrich, St. Louis, MO, USA). Stained skin sections were visualized with a BX53 microscope with a DP80 Digital Microscope Camera and CellSens Standard Software 3.1-Build 21199 (Olympus, Philadelphia, PA, USA) at 100-fold magnification [43].

### 2.5. Immunohistochemistry Staining for Aplha-Smooth Muscle Actin (aSMA)

Assessment of myofibroblast counts was performed as described previously [43]. In brief, for the detection of alpha-smooth muscle actin (aSMA)-positive myofibroblasts, skin sections were deparaffinized, followed by incubation with 5% horse serum in PBS for 1 h to block nonspecific binding and incubation with 3% H_2_O_2_ for 10 min to block endogenous peroxidase activity. The cells positive for aSMA in the lesional skin sections were detected by incubation with mouse monoclonal anti-aSMA antibody (1:1000, clone 1A4, Sigma-Aldrich, St. Louis, MO, USA) for three hours at room temperature. Irrelevant isotype-matched antibodies were used as controls. Horseradish peroxidase-labeled polyclonal rabbit anti-mouse antibodies (1:200, Dako, Glostrup, Denmark) were then used as secondary antibodies for incubation for one hour at room temperature. The skin sections were then counterstained with Mayer’s hematoxylin solution and visualized with a BX53 microscope with a DP80 Digital Microscope Camera and CellSens Standard Software v.3.1-Build 21199 (Olympus, Philadelphia, PA, USA) at 100-, 200-, and 400-fold magnification. The assessment of the number of myofibroblasts (at 200-fold magnification) was performed by two experienced examiners blinded to the treatment in the full thickness of the dermis at four sites in four consecutive skin sections of the lesional skin from each mouse. The mean value for each mouse was used [43].

### 2.6. Immunofluorescence Staining

Immunofluorescence staining was performed as described previously [45,46]. For the identification of cells expressing the phosphorylated Smad3 (pSmad3) [45], or TGF-β-receptor type I (TβRI) and II (TβRII) [46] in the lesional skin, deparaffinized sections were incubated with antigen retrieval solution (Dako. Glostrup, Denmark) for 15 min at 95 °C, followed by incubation with 5% horse serum in PBS for 1 h to block nonspecific binding. Phosphorylated Smad3-positive cells were detected by incubation with rabbit monoclonal anti-pSmad3 antibodies (phospho S423 + S425; 1:50, Abcam, Cambridge, UK) overnight at 4 °C [45]. TβRI- or TβRII-positive cells were detected by incubation with rabbit polyclonal anti-TβRI antibodies (PA5-32631; 1:50, Invitrogen, Carlsbad, CA, USA) or rabbit polyclonal anti-TβRII antibodies (AB186838; 1:100, Abcam, Cambridge, UK) overnight at 4 °C [46]. Irrelevant isotype-matched antibodies were used as controls. The skin samples were then incubated with polyclonal goat anti-rabbit Alexa Fluor 488 secondary antibodies (1:200, Abcam, Cambridge, UK) at room temperature for 1 h. The cell nuclei were stained using 4′,6-diamidino-2-phenylindole (DAPI, 1:1000, Invitrogen, Carlsbad, CA, USA) for 10 min at room temperature. Finally, the skin sections were mounted with Fluoromount aqueous mounting medium (Sigma-Aldrich, St. Louis, MO, USA) [45]. Images were captured at 400-fold magnification using appropriate fluorescence filters on a BX53 microscope with a DP80 Color lens camera using CellSens Standard software v.3.1-Build 21199 (Olympus, Philadelphia, PA, USA). The assessment of the number of TβRI-, TβRII-, and pSmad3-positive cells was performed by two experienced examiners blinded to the treatment at four sites in four consecutive skin sections of the lesional skin from each mouse. The count of positive cells was normalized to the number of cells and is presented as TβRI, TβRII-, and pSmad3-positive cell percentage. The mean value for each mouse was used [45].

### 2.7. Measurement of Inflammatory Cytokines/Chemokines in the Serum

The concentration of selected inflammatory cytokines and chemokines in the serum of mice was analyzed as described previously [47] by a commercially available Bio-Plex Pro™ Mouse Cytokine 23-plex Assay (BIO-RAD, Irvine, CA, USA) according to the manufacturer’s instructions. The Bio-Plex Pro™ Mouse Cytokine 23-plex Assay measures the concentration of 23 cytokines and chemokines: IL-1α, IL-1β, IL-2, IL-3, IL-4, IL-5, IL-6, IL-9, IL-10, IL12p40, IL-12p70, IL-13, IL-17A, eotaxin, granulocyte colony-stimulating factor (G-CSF), granulocyte-macrophage colony-stimulating factor (GM-CSF), interferon-γ (IFN-γ), keratinocytes-derived chemokine (KC, also known as chemokine (C-X-C) motif ligand 1 (CXCL1)), monocyte chemoattractant protein (MCP)-1 (CCL2), macrophage inflammatory proteins (MIP)-1α (CCL3) and MIP-1β (CCL4), regulated on activation/normal T cell expressed and secreted (RANTES, CCL5), and tumor necrosis factor (TNF). The absorbance of the Bio-Plex Pro™ Mouse Cytokine 23-plex Assay was evaluated at Luminex BIO-PLEX 200 System (Bio-Rad, Hercules, CA, USA) [47]. Samples were measured as duplicates, and the mean value was used. 

### 2.8. Safety of 17-DMAG in Mice

The safety of 17-DMAG in mice was assessed as described in previous studies of novel potential antifibrotic agents in a modified bleomycin model [27,28,36,37]. All studied mice were examined daily for any kind of distress, e.g., changes in physical activity, behavior, food and water consumption, quality of stools, and the quality and texture of their fur. Furthermore, the weight of the mice was assessed weekly using calibrated scales from the first bleomycin injection until sacrifice. During necropsy, internal organs were inspected for any macroscopically visible changes, e.g., formation of tumors, hemorrhages, presence of pus, or other significant pathologies [27,28,36,37].

### 2.9. Statistical Analysis

All analyses and graphs were conducted using GraphPad Prism 5 (v.5.02; GraphPad Software, La Jolla, CA, USA). Basic descriptive statistics (mean, standard error of the mean (SEM), skewness, and kurtosis) were computed for all variables, which were subsequently tested for normality using the Kolmogorov–Smirnov and Shapiro–Wilk tests. Differences between groups were analyzed by unpaired t-test with Welch’s correction or the Mann–Whitney U test. Statistical significance was set at *p* < 0.05. Data are presented as mean ± SEM. 

## 3. Results

### 3.1. Treatment with 17-DMAG Prevents Progression and May Induce Regression of Preestablished Bleomycin-Induced Skin Fibrosis

Using the modified bleomycin model of experimental dermal fibrosis, we investigated the effect of Hsp90 inhibitor 17-DMAG on the progression of dermal fibrosis, and of particular interest, the treatment of established skin fibrosis, since the 17-DMAG treatment was initiated after the development of dermal fibrosis. The efficacy of 17-DMAG was then compared to that of the established antifibrotic agent nintedanib.

**Figure 2:**
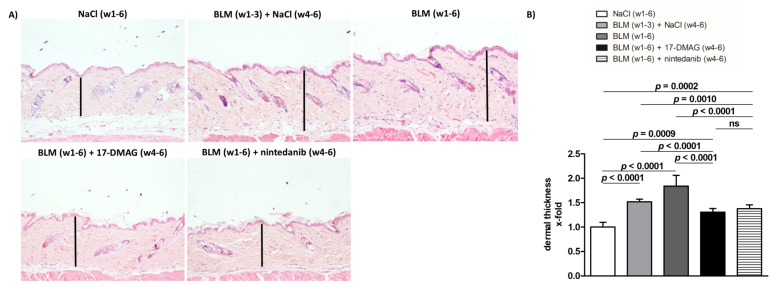
Treatment with 17-DMAG prevents further progression and may induce regression of dermal thickening induced by bleomycin. (**A**) Representative images of hematoxylin and eosin-stained skin sections are shown. Original magnification ×100. Vertical bars represent the dermal thickness. (**B**) Treatment with 17-DMAG prevents further progression and may induce regression of dermal thickening induced by bleomycin. The extent of the protective effects of 17-DMAG is comparable to the effect of the treatment with nintedanib. Columns represent the mean, and whiskers represent the standard error of the mean. w, week; NaCl, sodium chloride; BLM, bleomycin; 17-DMAG, 17-dimethylaminoethylamino-17-demethoxygeldanamycin (inhibitor of heat shock protein 90); ns, not significant (*p* ≥ 0.05); *n* = 8 mice in each group.

**Figure 3:**
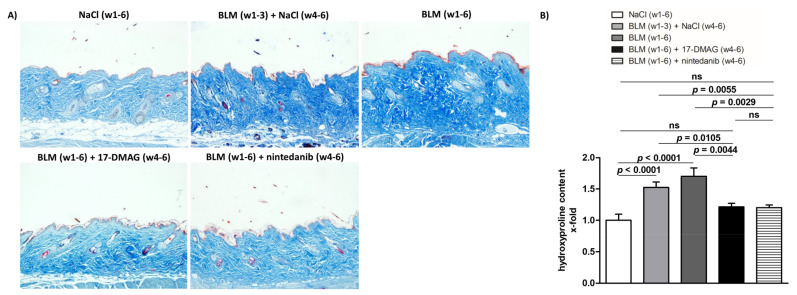
Treatment with 17-DMAG prevents further progression and may induce regression of collagen accumulation induced by bleomycin. (**A**) Representative images of Blue Masson’s Trichrome-stained skin sections are shown. Collagen bundles are stained blue. Original magnification ×100. (**B**) Treatment with 17-DMAG prevents further progression and may induce regression of collagen accumulation (analyzed by hydroxyproline content) induced by bleomycin. The extent of the protective effects of 17-DMAG is comparable to the effect of the treatment with nintedanib. Columns represent the mean and whiskers represent the standard error of the mean. w, week; NaCl, sodium chloride; BLM, bleomycin; 17-DMAG, 17-dimethylaminoethylamino-17-demethoxygeldanamycin (inhibitor of heat shock protein 90); ns, not significant (*p* ≥ 0.05); *n* = 8 mice in each group.

**Figure 4:**
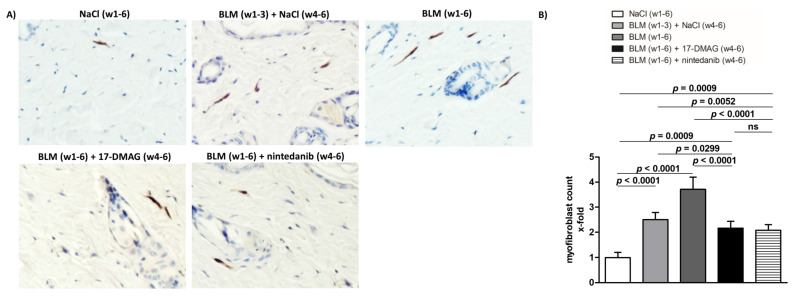
Treatment with 17-DMAG prevents further progression and may induce regression of proliferation of myofibroblasts induced by bleomycin. (**A**) Representative images of α-smooth muscle actin (aSMA)-stained skin sections are shown. aSMA-positive cells are stained brown, nuclei are counterstained blue by hematoxylin. Original magnification ×400. (**B**) Treatment with 17-DMAG prevents further progression and may induce regression of the proliferation of myofibroblasts induced by bleomycin. The extent of the protective effects of 17-DMAG is comparable to the effect of the treatment with nintedanib. Columns represent the mean and whiskers represent the standard error of the mean. w, week; NaCl, sodium chloride; BLM, bleomycin; 17-DMAG, 17-dimethylaminoethylamino-17-demethoxy-geldanamycin (inhibitor of heat shock protein 90); ns, not significant (*p* ≥ 0.05); *n* = 8 mice in each group.

Compared to mice treated with NaCl for six weeks (group 1), the challenge with bleomycin in the first three weeks (group 2) resulted in an expected development of skin fibrosis, manifested by an increase in dermal thickening by 51.5 ± 3.9% (*p* < 0.0001), hydroxyproline content by 52.4 ± 13.2% (*p* = 0.0014), and myofibroblast count by 150.3 ± 12.5% (*p* < 0.0001) (Figure 2, Figure 3 and Figure 4). Extended bleomycin treatment during the subsequent three weeks (group 3) led to further progression of skin fibrosis, with an additional increase in dermal thickening, hydroxyproline content, and myofibroblast count (*p* < 0.05 for all, compared to group 2; *p* < 0.001 for all, compared to group 1) (Figure 2, Figure 3 and Figure 4). 

Treatment with 17-DMAG (group 4) during the last three weeks of bleomycin treatment inhibited this progression despite concurrent bleomycin injections (Figure 2, Figure 3 and Figure 4). Compared with vehicle-treated mice challenged with bleomycin for six weeks (group 3), 17-DMAG treatment (group 4) prevented the increase in skin thickness by 53.5 ± 8.2% (*p* < 0.0001) (Figure 2A,B), hydroxyproline content by 48.8 ± 14.4% (*p* = 0.0044) (Figure 3B), and myofibroblast count by 154.5 ± 19.8% (*p* < 0.0001) (Figure 4A,B). Thus, treatment with 17-DMAG effectively prevented the progression of established dermal fibrosis induced by bleomycin.

Furthermore, the extent of dermal fibrosis upon 17-DMAG treatment (group 4) decreased even below the pretreatment levels represented by mice treated with bleomycin for three weeks followed by NaCl for three weeks (group 2): skin thickness decreased by 21.1 ± 3.3% (*p* < 0.0001) (Figure 2A,B), hydroxyproline content by 30.8 ± 10.4% (*p* = 0.0105) (Figure 3B), and myofibroblast count by 33.5 ± 13.9% (*p* = 0.0299) (Figure 4A,B). Thus, treatment with 17-DMAG may induce regression of established dermal fibrosis induced by bleomycin.

Interestingly, the decreased extent of fibrosis in the 17-DMAG-treated mice (group 4), as demonstrated by the abovementioned skin thickness, hydroxyproline content, and myofibroblast count, was comparable to that observed in mice treated with the established antifibrotic agent nintedanib (group 5) (*p* = 0.0919, *p* = 0.8551, *p* = 0.4776, respectively) (Figure 2, Figure 3 and Figure 4).

### 3.2. 17-DMAG Reduces the Activation of TGF-β Smad Signaling in Bleomycin-Induced Dermal Fibrosis

**Figure 5:**
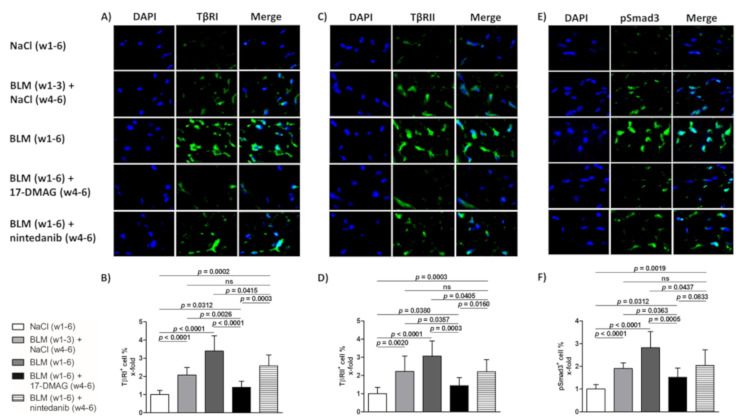
Treatment with 17-DMAG inhibits the TGF-β/Smad signaling induced by bleomycin. Representative images of TβRI- (**A**), TβRII- (**C**), and phosphorylated Smad3 (pSmad3)-stained skin sections are shown (**E**). TβRI-, TβRII-, and pSmad3-positive cells are stained green, and nuclei are stained blue by DAPI. Original magnification ×400. Treatment with 17-DMAG decreased the accumulation of TβRI- (**B**), TβRII- (**D**), and pSmad3 (**F**) induced by bleomycin. Columns represent the mean and whiskers represent the standard error of the mean. w, week; NaCl, sodium chloride; BLM, bleomycin; 17-DMAG, 17-dimethylaminoethylamino-17-demethoxygeldanamycin (inhibitor of heat shock protein 90); TβRI, TGF-β receptor type I; TβRII, TGF-β receptor type II; ns, not significant (*p* ≥ 0.05); *n* = 8 mice in each group.

In addition, given the Hsp90-mediated stabilization of TβRI and TβRII [25], the abrogation of profibrotic effects of TGF-β upon 17-DMAG treatment demonstrated in our previous study [26], and the suppression of TGF-β Smad signaling upon Hsp90 inhibition demonstrated by Noh et al. [48], we aimed to investigate the impact of 17-DMAG on the activation of TGF-β signaling by assessing the expression of TGF-β type I and II receptors and the intracellular pathway mediated by Smad3. Compared to mice treated with NaCl for six weeks (group 1), we observed an expected activation of TGF-β signaling with an increased percentage of TβRI-, TβRII-, and pSmad3-positive cells in the lesional skin upon challenge with bleomycin for three weeks (group 2, by 108.3 ± 16.5%, *p* < 0.0001; 122.2 ± 29.9%, *p* = 0.0020; 90.6 ± 13.2%, *p* < 0.0001, respectively) or six weeks (group 3, by 239.9 ± 30.7%, *p* < 0.0001; 206.4 ± 32.0%, *p* < 0.0001; 181.9 ± 26.1%, *p* < 0.0001, respectively) (Figure 5A–F). Indeed, treatment with 17-DMAG (group 4) significantly decreased the percentage of TβRI-, TβRII-, and pSmad3-positive cells (by 199.5 ± 31.8%, *p* < 0.0001; 163.0 ± 33.7%, *p* = 0.0003; 130.3 ± 28.4%, *p* = 0.0005 compared to group 3, and by 67.9 ± 18.6%, *p* = 0.0026; 78.8 ± 33.9%, *p* = 0.0357; 39.0 ± 16.9%, *p* = 0.0363 compared to group 2, respectively), as well as the intensity of staining (Figure 5A–F). Thus, treatment with 17-DMAG strongly downregulated TGF-β/pSmad3 signaling in the lesional skin of mice challenged with bleomycin to below pretreatment levels. Nintedanib treatment (group 5) demonstrated mild reduction in TGF-β/pSmad3 signaling upon bleomycin challenge (*p* < 0.05 for all three outcomes compared to group 3).

### 3.3. 17-DMAG Treatment Reduces Local and Systemic Inflammation in Bleomycin-Induced Dermal Fibrosis

**Figure 6:**
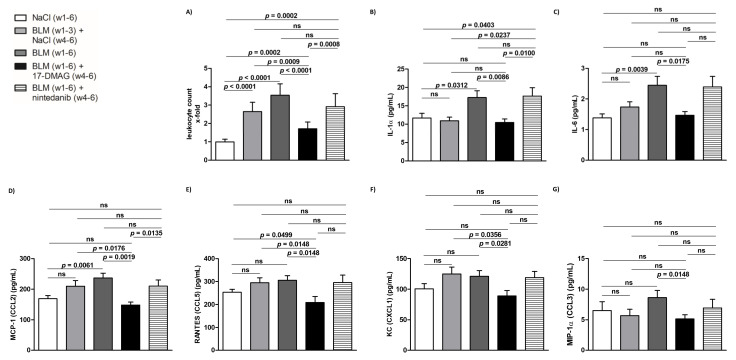
Treatment with 17-DMAG strongly downregulates the local and systemic proinflammatory response induced by bleomycin. Treatment with 17-DMAG significantly reduced the number of leukocytes infiltrating the lesional skin (**A**), and the serum levels of interleukin (IL)-1α (**B**), IL-6 (**C**), monocyte chemoattractant protein-1 (MCP-1, CCL2) (**D**), regulated on activation/normal T cell expressed and secreted (RANTES, CCL5) (**E**), keratinocytes-derived chemokine (KC, CXCL1) (**F**), and macrophage inflammatory proteins-1α (MIP1α, CCL3) (**G**). Columns represent the mean and whiskers represent the standard error of the mean. w, week; NaCl, sodium chloride; BLM, bleomycin; 17-DMAG, 17-dimethylaminoethylamino-17-demethoxygeldanamycin (inhibitor of heat shock protein 90); ns, not significant (*p* ≥ 0.05); *n* = 8 mice in each group.

The mouse model of bleomycin-induced experimental dermal fibrosis mimics the early stages of SSc, characterized by perivascular inflammatory infiltrates in the dermis containing leukocytes, including T and B lymphocytes, macrophages, eosinophils, and mast cells, which stimulate fibroblast activation and collagen synthesis by releasing profibrotic cytokines and growth factors [2,7,34,49]. Given the crucial role of Hsp90 in the innate and adaptive immune system [16,17], and to investigate whether 17-DMAG affects the outcome of bleomycin-induced dermal fibrosis partially by regulating inflammatory infiltration, we further quantified the number of leukocytes in the lesional skin. Compared to mice treated with NaCl for six weeks (group 1), we observed expectedly elevated numbers of leukocytes infiltrating the lesional skin upon challenge with bleomycin for three weeks (group 2, by 164.4 ± 18.7%, *p* < 0.0001) or six weeks (group 3, by 253.9 ± 22.6%, *p* < 0.0001) (Figure 6A). Treatment with 17-DMAG (group 4) significantly decreased the leukocyte count (by 182.9 ± 25.5%, *p* < 0.0001 compared to group 3, and by 93.4 ± 22.2%, *p* = 0.0009 compared to group 2) (Figure 6A). Thus, treatment with 17-DMAG strongly reduced the inflammatory infiltration in the lesional skin of mice challenged with bleomycin to below pretreatment levels. Nintedanib treatment (group 5) demonstrated only a mild reduction in the inflammatory infiltration in the lesional skin upon bleomycin challenge (by 62.6 ± 33.3%, *p* = 0.0803 compared to group 3) (Figure 6A).

Given the documented systemic manifestations of prolonged subcutaneous administration of bleomycin in mice [34,39,50], we determined the systemic levels of selected proinflammatory cytokines and chemokines which have been implicated in the pathophysiology of SSc [1,2,7,29], in order to further assess the potential regulation of inflammatory infiltration and the associated fibroblast activation in the lesional skin mediated by Hsp90. Compared to NaCl-treated mice (group 1), in mice challenged with bleomycin for six weeks (group 3), we found an increase in serum levels of IL-1α, IL-6, and MCP-1 (CCL2), which was statistically significant (*p* < 0.05 for all), and a trend to increased serum levels of CCL5, CXCL1, and CCL3, which did not reach the level of statistical significance (*p* = 0.074, *p* = 0.128, *p* = 0.130, respectively) (Figure 6B–G). Nevertheless, 17-DMAG treatment (group 4) resulted in a significant decrease (*p* < 0.05 for all, compared to group 3) in serum levels of all these proinflammatory cytokines/chemokines to levels comparable to those observed in the NaCl-treated mice (group 1) (Figure 6B–G). Thus, treatment with 17-DMAG strongly downregulated the systemic proinflammatory response induced by extended subcutaneous administration of bleomycin in experimental dermal fibrosis. No differences were observed in the remaining cytokines or chemokines measured by the Bio-Plex Pro™ Mouse Cytokine 23-plex Assay (*p* > 0.05 for all). No differences in serum levels of any of these cytokines/chemokines were demonstrated in mice treated with nintedanib (group 5) upon bleomycin challenge (Figure 6B–G).

## 4. Discussion

In this study, we demonstrate for the first time that treatment with 17-DMAG, an Hsp90 inhibitor, effectively prevents progression and may induce regression of established experimental dermal fibrosis induced by bleomycin. The extent of reduction of dermal fibrosis mediated by 17-DMAG is comparable to the effects of the treatment with nintedanib, an established antifibrotic agent. The antifibrotic effects of 17-DMAG in this modified bleomycin model are not only mediated by direct inhibition of fibroblast activation via the downregulation of TGF-β/Smad signaling, but also indirectly via a potent suppression of the local and systemic inflammatory response. This dual mode of action translates into potent antifibrotic effects of 17-DMAG.

A large body of evidence has demonstrated the crucial role of Hsp90 in tumorigenesis and metastasis, particularly by maintaining homeostasis of several oncogenic proteins, including a number of transcription factors and kinases [18,19,22]. Thus, over the last two decades, several Hsp90 inhibitors have been tested in clinical trials, predominantly in solid tumors and hematological malignancies [18,30,33]. 17-DMAG is a second-generation inhibitor of Hsp90 characterized by higher water solubility, improved bioavailability, reduced toxicity, and higher therapeutic efficacy than its predecessors [32]. It suppresses the ATPase activity of Hsp90 and consequently leads to misfolding, ubiquitylation, and degradation of the client proteins of Hsp90 by the proteasome [31,32].

Compared to the well-documented role of Hsp90 in cancer, the understanding of the role of Hsp90 in tissue fibrosis is very limited. Hsp90 was upregulated in the lung biopsies of patients with idiopathic pulmonary fibrosis and in the isolated fibroblasts from the fibrotic lung lesions. Furthermore, inhibition of Hsp90 by 17-N-allylamino-17-demethoxy-geldanamycin (17-AAG) efficiently reduced TGF-β-driven activation of fibroblasts and production of ECM in vitro and attenuated progression of established fibrosis in a mouse model of pulmonary fibrosis [51]. More recently, five other studies have confirmed potent antifibrotic effects of various Hsp90 inhibitors, including 17-DMAG, in several preclinical models of pulmonary fibrosis [52,53,54,55,56]. Similarly, the treatment with 17-AAG blocked the TGF-β-induced production of ECM in renal fibroblasts in vitro and suppressed renal fibrosis in a murine model of unilateral ureteral obstruction [48]. Inhibition of Hsp90 with an engineered protein inhibitor reduced the TGF-β-mediated profibrotic events in cardiac fibroblasts in vitro and ameliorated experimental murine myocardial fibrosis induced by angiotensin-II [57]. Our recent study on the role of Hsp90 in SSc demonstrated an increased expression of Hsp90 in the SSc skin and dermal fibroblasts and in experimental dermal fibrosis in a TGF-β-dependent manner [26]. Treatment with 17-DMAG effectively abrogated the profibrotic effects of TGF-β in cultured dermal fibroblasts and prevented the development of experimental dermal fibrosis in three different murine models of SSc [26]. 

In line with these findings, in this study, we provide evidence that treatment with 17-DMAG not only effectively prevents further progression, but may also promote regression of established experimental dermal fibrosis induced by bleomycin. However, given the limited capacity of group 2 to precisely represent the pretreatment level of fibrosis, regression of the established experimental dermal fibrosis induced by 17-DMAG treatment should be interpreted with caution. Compared to vehicle-treated mice, treatment with 17-DMAG decreased dermal thickening, hydroxyproline content, and myofibroblast count to below pretreatment levels. Given the proven efficacy of several potential antifibrotic therapies in the preclinical model of established dermal fibrosis [8], we selected nintedanib as a positive treatment control. Nintedanib is a small-molecule competitive inhibitor of nonreceptor tyrosine kinases (nRTKs), such as lymphocyte-specific protein tyrosine kinase (Lck), tyrosine-protein kinase Lyn (Lyn), and Src. It also inhibits receptor tyrosine kinases (RTKs), such as platelet derived growth factor (PDGF) receptor α and β, fibroblast growth factor (FGF) receptor 1–3, vascular endothelial growth factor (VEGF) receptor 1–3, and fms-like tyrosine kinase 3 (FLT-3) [58]. Moreover, nintedanib has previously demonstrated efficacy in the regression of preestablished experimental dermal fibrosis induced by bleomycin [28], as well as in other preclinical models of tissue fibrosis [28,42,58], and was recently approved by Food and Drug Administration (FDA) for slowing the rate of decline in lung function in adults with SSc-associated interstitial lung disease (ILD) [59]. Interestingly, in this study, the extent of the antifibrotic effects of 17-DMAG treatment was comparable to that of nintedanib treatment. Previous studies on nintedanib in preclinical models of tissue fibrosis have attributed its antifibrotic effects to several possible mechanisms. In vitro, direct antifibrotic effects of nintedanib were mediated by the inhibition of PDGF- and TGF-β-induced proliferation, migration and activation of fibroblasts, as well as myofibroblast differentiation and collagen release [28]. In vivo, possible indirect mechanisms include the anti-inflammatory effects mediated by the inhibition of Lck and Lyn kinases [28], or by inhibiting the alternative activation of macrophages, along with decreased serum levels of M-CSF and VEGF in an Fra-2 mouse model [42]. In our present study, in contrast to 17-DMAG, the antifibrotic effects of nintedanib were not associated with major alterations in serum levels of proinflammatory cytokines or chemokines. 

In addition to the findings from our previous study [26], the antifibrotic effects of 17-DMAG observed in this study were mediated by the inhibition of TGF-β/Smad signaling, as was evidenced by the decreased percentage of TβRI-, TβRII-, and pSmad3-positive cells to below pretreatment levels. Our findings are in line with previously published observations in experimental renal and myocardial fibrosis [48,57]. Noh et al. demonstrated that 17-AAG treatment suppressed TGF-β-induced Smad signaling in renal fibroblasts via a mechanism dependent on proteasome-mediated degradation of TβRII [48]. Caceres et al. showed that inhibition of Hsp90 in myocardial fibroblasts resulted in the disruption of the TGFβRI-Hsp90 complex and a reduction in TGF-β/Smad signaling [57]. Nevertheless, the detailed mechanisms of the TGF-β signaling suppression by 17-DMAG in bleomycin-induced dermal fibrosis have yet to be elucidated by further studies.

Given the documented role of Hsp90 in the innate and adaptive immune system [16,17], we aimed to examine whether the effect of 17-DMAG on bleomycin-induced dermal fibrosis is partially mediated by the regulation of the immune response. The early stages of SSc are characterized by increased number and activation of inflammatory cells infiltrating the involved skin, which release proinflammatory and profibrotic cytokines that further stimulate the production of ECM in resident fibroblasts [2,7,49]. Similar to SSc, repeated subcutaneous administration of bleomycin in the experimental model of dermal fibrosis increases the number and activation of leukocytes infiltrating the lesional skin [34,60]. Our results demonstrate that 17-DMAG treatment significantly reduced the number of leukocytes infiltrating the lesional skin challenged with bleomycin, even to pretreatment levels. Our findings are in agreement with previous studies demonstrating anti-inflammatory effects of Hsp90 inhibitors, which significantly improved the clinical course in animal models of several autoimmune inflammatory diseases, such as autoimmune encephalomyopathy, rheumatoid arthritis, systemic lupus erythematosus-like autoimmune disease, and epidermolysis bullosa acquisita [61,62,63,64]. In addition, our recently published study demonstrated elevated systemic levels of Hsp90 in SSc patients, particularly in those with elevated C-reactive protein levels [65].

To further evaluate the Hsp90-mediated regulation of inflammatory infiltration and the associated fibroblast activation in the lesional skin challenged with bleomycin, we assessed the systemic levels of selected proinflammatory cytokines and chemokines, which represent the links between the immune response and the development of fibrosis in SSc [7,29]. Prolonged subcutaneous administration of bleomycin in the murine model of experimental dermal fibrosis also results in systemic manifestations, in particular, lung fibrosis exhibiting thickened alveolar walls with cellular infiltrates [60]. However, intratracheal (or intranasal) administration (single-dose or modified repeated-dose protocol) of bleomycin in rodents represents the golden standard in preclinical models of experimental lung fibrosis [66], and the antifibrotic effects of 17-DMAG in such models have been previously demonstrated [55]. Therefore, the impact of 17-DMAG on lung fibrosis was not assessed in this study. Nevertheless, in bleomycin-challenged vehicle-treated mice, we demonstrate a significant increase in serum levels of IL-1α, IL-6, and MCP-1 (CCL2), and a trend to increased serum levels of RANTES (CCL5), KC (CXCL1), and MIP-1α (CCL3). Treatment with 17-DMAG significantly reduced the serum levels of all these proinflammatory cytokines and chemokines to an extent comparable to the levels of NaCl-challenged mice. The role of these cytokines and chemokines in SSc has already been well established [1,2,7,29]. Expression of IL-1α is spontaneously increased in SSc fibroblasts and additionally induces expression of IL-6, PDGF, and the fibrogenic phenotype of SSc fibroblasts [67,68]. IL-6 has a crucial role in the development of fibrosis and inflammation in SSc, substantiated by its increased levels in serum, skin, SSc fibroblasts, and peripheral blood mononuclear cells (PBMCs). Moreover, inhibition of IL-6 by tocilizumab demonstrated promising effects in stabilizing SSc-ILD in a randomized controlled trial [7,69,70]. MCP-1 (CCL2) is one of the most robustly described chemokines with an established role in mediating fibrotic immune responses in SSc, and its levels are increased in SSc serum, skin, and bronchoalveolar lavage (BAL) fluid [29]. Elevated levels of RANTES (CCL5) and/or MIP-1α (CCL3) have been found in SSc serum, BAL fluid, skin, and PBMCs [29]. Increased serum levels of CXCL1 were associated with deteriorated lung function in SSc patients [71]. However, the potential impact of 17-DMAG in the preclinical models of SSc on serum levels of other cytokines and chemokines, which play vital roles in the pathogenesis of SSc and the progression of fibrosis, such as TGF-β [3] and IL-8 [71], needs to be elucidated by further investigation. Several studies involving different Hsp90 inhibitors have already demonstrated antifibrotic effects mediated by the inhibition of the TGF-β pathway in experimental models of lung fibrosis [51,52,53,54,55,56]. Interestingly, in the study by Sibinska et al. [55], only the lower dose of 17-DMAG (i.e., 10 mg/kg vs. 25 mg/kg) significantly decreased both the bronchoalveolar lavage fluid and serum levels of TGF-β in the bleomycin-induced pulmonary fibrosis. Similarly, inhibition of Hsp90 reduced local or systemic levels of IL-8 in experimental models of various inflammatory and tumorous conditions [72,73,74,75,76]. However, further studies are required to elucidate the mechanisms underlying the suppression of local and systemic inflammatory response mediated by 17-DMAG, as well as the potential link between the humoral or cellular immune response and the progression of dermal fibrosis induced by bleomycin.

The treatment with 17-DMAG was well tolerated without obvious signs of toxicity on clinical examination, or on gross necropsy. No reduction in weight was detected in 17-DMAG-treated mice compared to mice injected subcutaneously with bleomycin only. Nevertheless, data on the most common dose-limiting toxicities of 17-DMAG are already available from seven clinical trials in hematological malignancies and solid tumors, and symptoms include fatigue, nausea, vomiting, diarrhea, anorexia, and liver enzyme disturbances [32]. Therefore, our findings on the efficacy of 17-DMAG in the treatment of established experimental dermal fibrosis need to be validated by further studies, preferably using the more recently developed Hsp90 inhibitors with improved safety profile [33].

## 5. Conclusions

We demonstrate that treatment with 17-DMAG, an Hsp90 inhibitor, effectively prevented further progression and may induce regression of established bleomycin-induced dermal fibrosis with a comparable outcome to that of nintedanib, a well-established antifibrotic agent. Antifibrotic effects of 17-DMAG were mediated by direct inhibition of fibroblast activation via the suppression of TGF-β/Smad signaling and indirectly by reduction of the local and systemic inflammatory response induced by bleomycin. The treatment with 17-DMAG was well tolerated without obvious clinical signs of toxicity. Our findings further support the vital role of Hsp90 in the pathophysiology of SSc, thus providing a potential target for the treatment of fibrosis with translational implications due to the availability of several Hsp90 inhibitors in clinical trials for other indications. In addition, given our recently published results [65], which demonstrated increased systemic levels of Hsp90 in SSc patients, their association with systemic inflammation, skin and lung involvement, and their ability to predict the treatment response in SSc-ILD, Hsp90 could be a potential biomarker for stratification of SSc patients suitable for targeted antifibrotic treatment.

## Data Availability

Individual anonymized data will not be shared. Pooled study data, protocol, or statistical analysis plans can be shared upon request at tomcik@revma.cz.

## Abbreviations

Hsp90	heat shock protein 90
SSc	systemic sclerosis
17-DMAG	17-dimethylaminoethylamino-17-demethoxy-geldanamycin
pSmad3	phosphorylated small mothers against decapentaplegic homolog 3
ECM	extracellular matrix
TGF-β	transforming growth factor-beta
aSMA	alpha-smooth muscle actin
TβRI	transforming growth factor-beta type I
TβRII	transforming growth factor-beta type II
PDGF	platelet-derived growth factor
IL	interleukin
MCP-1	monocyte chemoattractant protein 1
CCL	CC chemokine
Hsp	heat shock proteins
TNF	tumor necrosis factor
Src	proto-oncogene tyrosine-protein kinase Src
C57BL/6	laboratory strain of mice referred to as black 6
NaCl	sodium chloride
PBS	phosphate buffered saline
nRTKs	nonreceptor tyrosine kinases
Lck	lymphocyte-specific protein tyrosine kinase
Lyn	tyrosine-protein kinase Lyn
RTKs	receptor tyrosine kinases
FGF	fibroblast growth factor
VEGF	vascular endothelial growth factor
FLT-3	fms-like tyrosine kinase 3
FDA	Food and Drug Administration
ILD	interstitial lung disease
AZV	Czech Health Research Council
MSMT	Ministry of Education, Youth and Sports of the Czech Republic
3Rs	Replacement of animals by alternatives wherever possible, Reduction in number of animals used, and Refinement of experimental conditions and procedures to minimize the harm to animals
HCl	hydrogen chloride
pH	potential of hydrogen
NaOH	sodium hydroxide
H2O2	hydrogen peroxide
HRP	horseradish peroxidase
DAPI	4’,6-diamidino-2-phenylindole
G-CSF	granulocyte colony-stimulating factor
GM-CSF	granulocyte-macrophage colony-stimulating factor
IFN-γ	interferon-γ
KC	keratinocytes-derived chemokine
CXCL1	chemokine C-X-C motif ligand 1
MIP-1α	macrophage inflammatory protein-1α, also referred to as CCL3
MIP-1β	macrophage inflammatory protein-1β, also referred to as CCL4
RANTES	regulated on activation/normal T cell expressed and secreted, also referred to as CCL5
SEM	standard error of the mean
P	*p*-value
17-AAG	17-N-allylamino-17-demethoxy-geldanamycin
PBMCs	peripheral blood mononuclear cells
BAL	bronchoalveolar lavage, w: week
BLM	bleomycin, NINT: nintedanib
N	number of mice
s.c.	subcutaneous
i.p.	intraperitoneal
p.o.	peroral
