# Correction: Štorkánová et al. Inhibition of Hsp90 Counteracts the Established Experimental Dermal Fibrosis Induced by Bleomycin. *Biomedicines* 2021, *9*, 650

**DOI:** 10.3390/biomedicines11102736

**Published:** 2023-10-09

**Authors:** Hana Štorkánová, Lenka Štorkánová, Adéla Navrátilová, Viktor Bečvář, Hana Hulejová, Sabína Oreská, Barbora Heřmánková, Maja Špiritović, Radim Bečvář, Karel Pavelka, Jiří Vencovský, Jörg H. W. Distler, Ladislav Šenolt, Michal Tomčík

**Affiliations:** 1 Institute of Rheumatology, 12800 Prague, Czech Republic; storkanova@revma.cz (H.Š.); storkanoval@revma.cz (L.Š.); navratilova@revma.cz (A.N.); becvarv@revma.cz (V.B.); hulejova@revma.cz (H.H.); oreska@revma.cz (S.O.); spiritovic@revma.cz (M.Š.); becvar@revma.cz (R.B.); pavelka@revma.cz (K.P.); vencovsky@revma.cz (J.V.); senolt@revma.cz (L.Š.); 2 Department of Rheumatology, First Faculty of Medicine, Charles University, 12800 Prague, Czech Republic; 3 Department of Physiotherapy, Faculty of Physical Education and Sport, Charles University, 16252 Prague, Czech Republic; hermankova@revma.cz; 4 Department of Internal Medicine III and Institute for Clinical Immunology, University of Erlangen-Nuremberg, 91054 Erlangen, Germany; joerg.distler@uk-erlangen.de

## Error in Figure

In the original publication [1], there was a mistake in Figure 4 as published. The representative picture of the immunohistochemistry staining for aSMA of the group entitled BLM (w1-6) + nintedanib (w4-6) was erroneously selected from a different group of mice. The corrected Figure 4 appears below. The authors state that the scientific conclusions are unaffected. This correction was approved by the Academic Editor. The original publication has also been updated.

## Figures and Tables

**Figure 4:**
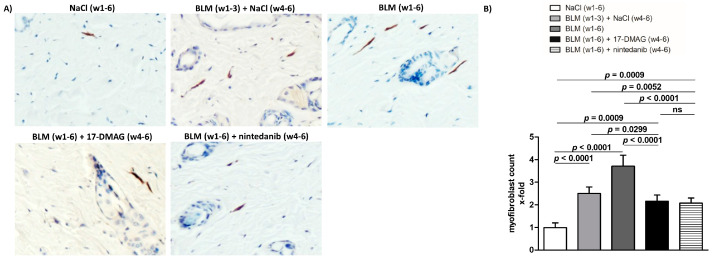
Treatment with 17-DMAG prevents further progression and may induce regression of proliferation of myofibroblasts induced by bleomycin. (**A**) Representative images of α-smooth muscle actin (aSMA)-stained skin sections are shown. aSMA-positive cells are stained brown, nuclei are counterstained blue by hematoxylin. Original magnification ×400. (**B**) Treatment with 17-DMAG prevents further progression and induces regression of the proliferation of myofibroblasts induced by bleomycin. The extent of the protective effects of 17-DMAG is comparable to the effect of the treatment with nintedanib. Columns represent the mean, and whiskers represent the standard error of the mean. w, week; NaCl, sodium chloride; BLM, bleomycin; 17-DMAG, 17-dimethylaminoethylamino-17-demethoxygeldanamycin (inhibitor of Heat shock protein 90); ns, not significant (*p* ≥ 0.05); *n* = 8 mice in each group.
